# Child disability as a family issue: a study on mothers’ and fathers’ health in Italy

**DOI:** 10.1093/eurpub/ckad168

**Published:** 2023-10-05

**Authors:** Nicoletta Balbo, Danilo Bolano

**Affiliations:** Department of Social and Political Sciences, Bocconi University, Milan, Italy; Dondena Centre, Bocconi University, Milan, Italy; Dondena Centre, Bocconi University, Milan, Italy

## Abstract

**Background:**

Disability does not simply affect the health status of the individual who directly experiences that condition, but it has important consequences on the health and well-being of the other family members as well. Focusing on Italy, an extremely interesting test-bed due to its strong familialist welfare regime, we show significant spillover effects of children’s disability on parental health and well-being.

**Methods:**

We use data from a nationally representative household survey on almost 13 000 mothers and fathers and adopt a multivariate regression setting providing evidence that the disability of a child is negatively associated with parents’ health and life satisfaction.

**Results:**

Parents of a disabled child report lower levels of general and mental health, as well as lower levels of well-being compared with parents with a healthy child. Strong heterogeneity by gender and socio-economic characteristics is observed, with mothers being more affected by the disability status of the child than fathers. The estimated coefficients suggest that education remains an important protective factor even for parents of a disabled child.

**Conclusion:**

This study claims and documents that child disability is an overlooked source of health disadvantage for parents. Such disadvantage is especially relevant for mothers and lower-educated parents, evidence that suggests the importance of taking an intersectional approach to study health disparities.

## Introduction

Individuals with a disability are the world’s largest minority. One billion people worldwide live with some form of disability, which is ∼15% of the global population. Disability not only impacts the lives of those who are directly affected, but it also has important spillover effects on family members.^1^ While most of the studies focus on disability among older adults and the relative care burden,^2–5^ this study shifts the attention to an understudied dimension of disability, namely child disability, looking at it as an overlooked source of health disadvantage and a shaping factor for the life of their parents.

A good reason not to conflate disability and aging, is even just the presence of nearly 240 million children with disabilities in the world.^6^ Focusing on the European Union, ∼4% of individuals under the age of 16 have some disabilities,^7^ and over 15 million school-age children have been identified as having special educational needs.^8^ These disabilities limit children in their everyday activities and impact the lives of their families in myriad ways. In the present paper, we investigate the spillover effects of child disability on parents’ health, taking an intersectional perspective that allows us to examine how health disparities come from several sources of inequality, such as gender and socio-economic status (SES), next to child disability.

This work aims at documenting the importance of investigating mechanisms of inequality using a reversed and unexplored perspective with respect to conventional wisdom: the source of disadvantage is not a parental characteristic affecting offspring’s health, as highlighted by the literature emphasizing the importance of the first thousand days of life,^9^ but a child’s condition negatively impacting parents’ health outcomes. Such a perspective has so far been adopted only by a handful number of studies to investigate the effect of: (i) adolescent depressive symptoms on parental depressive symptoms,^10^^,^^11^ showing ‘upward’ intergenerational transmission of mental-ill health and (ii) life course changes of children (aged 15+ years) on parents’ well-being.^12^

There is some scattered evidence suggesting that being confronted with the disability of a child may substantially affect the life and well-being of family members (mainly parents), and shape their social, demographic, and economic trajectories. A relevant weakness of the existing literature is that many studies rely on data from small convenience samples, often focusing on one specific type of child disability, and rarely have sufficient data to consider other, crucial factors. All this makes any generalizations to the population level, as well as to a broader scientific setting, unfeasible. Notable exceptions of population-based studies can be found in the USA^13^^,^^14^ and Canada,^15^^,^^16^ while in European studies a population approach is almost exclusively devoted to child disability and parental employment.^17–19^

Using a nationally representative household survey of Italian families and applying a multivariate regression model approach, we aim at identifying the relationship between child disability and parental health. We will consider both general health of health (self-rated level of health), mental health (from SF-36 0–100 score) and well-being (life satisfaction 0–10 score). By studying all these different health dimensions, we can uncover how child disability is associated with different spheres of health and well-being of these parents.

We focus on the Italian context, where evidence on the topic is virtually absent, although it is an extremely interesting test-bed because of its strong familialist welfare regime. Italy is a country where the family, and in most cases the mother, is the main responsible for the care of children or non-independent/sick family members. Within this context characterized by a rather unequal gender system,^20^ we aim at investigating differences in maternal and paternal health and well-being using an intersectional approach that jointly considers gender and child disability as two potential sources of disadvantage. Beliefs and norms about gender differences combined with structurally unequal relationships lead men and women to enact gendered divisions of tasks in the household. Therefore, we expect to find gender-stratified consequences of child disability on Italian parents, with mothers of a disabled child reporting lower levels of health, due to higher care burden, likely being the mother, the main caregiver and norms of self-sacrifice.^21^

In line with recent work on mothers only in the USA,^22^ we also envision parental socio-economic status (SES) to work as an important protective factor against health risks due to child disability, because it likely translates into higher economic, social, and cultural capital that can help parents to deal with the increased costs, care burden, stigma and bureaucratic challenges that they have to face.

This study contributes to the existing literature on public health and social determinants of health by documenting the importance of child disability as an almost neglected albeit relevant factor shaping parents’ health condition, although the reverse direction of causation cannot be excluded. It does so, by using, in an understudied national context that is Italy, a population approach necessary for adjudicating whether contradictory findings are driven by sampling biases, selection processes or suggestive of legitimately differential effects by population subgroup. Moreover, such an approach enables us to compare parents with a disabled child with their counterpart with a healthy child, and to control for relevant socio-economic factors, that may act as confounders in the relationship under study. Ultimately, we believe the importance of studying extreme cases to learn about the general: the analysis of extremely frail families, such as the ones with a disabled child, can shed further light on the more general functioning of families when confronted with adverse events and their relative spillover effects on health.

### Child disability and parental health

Studies of children’s disability and family members’ health show mixed findings. Most studies report adverse effects on parents’ health.^15^^,^^23^ However, there is also some emphasis on how the positive impact of having a child with a disability, which consists of positive emotions such as love, pride and happiness, may translate into increased parental well-being.^24^ Along the same lines, other studies suggest that having a child with a disability is associated with higher frailty in terms of mental health, and emotional stress for parents;^25^^,^^26^ at the same time, a few studies highlight the ability of such families to adjust and develop coping strategies and resilience.^27^^,^^28^ Therefore, we aim at studying how child disability influences parents’ health considering different aspects of their well-being, to shed further light on which health dimensions are affected the most by the disability of a child. We focus first on self-rated health, which has been shown to be a good proxy also for physiological issues.^29^ Then, we decompose the different aspects of an individual’s general health by specifically examining the relationship between child disability and mental health, as well as the association between the former and life satisfaction. By distinguishing the different health components, we can better investigate whether we observe higher mental distress likely deriving from the heightened care burden, the anxiety and stigma or, rather, we find evidence for the ‘disability paradox’.^30^ This theory claims that disabled individuals tend to perceive a higher quality of life than non-disabled individuals because of their lower expectations and structural limitations. However, it is an open question—not yet empirically tested—whether that applies to the parents well.

The envisioned health disparities between parents with and without a disabled child are likely to be moderated by the gender and the socio-economic status (SES) of the parents. Existing literature has shown that mothers are likely to bear the higher costs in terms of the health of child disability.^31^ Very little is known about fathers because most existing studies on the topic focus on mothers only.^15^ As far as SES is concerned, there is sparse evidence that the lack of economic resources and poverty can explain the worse health status of parents with a disabled child,^32^ not only because of the increased costs that they have to bear but also because the majority of disabled children are observed in low SES families.^33^ However, what is missing, and what we aim to do in this study, is to systematically examine the moderating role of gender and SES in the relationships between child disability and three different health outcomes: general health, mental health and life satisfaction. In this way we can uncover whether the health disadvantage related to child disability is particularly relevant for specific social groups in the populations, likely mothers and low SES families.

## Data

Data are drawn from a nationally representative repeated cross-sectional survey ‘Aspects of Daily Life—AVQ’ administered to ∼25 000 households residing in Italy. AVQ is appropriate for this study as: (i) it is nationally representative allowing for a population approach rather than focusing on a convenient sample as done in other studies on disability and (ii) each member living in the household replied to the questionnaire. Each measure is then self-reported, therefore eliminating any potential bias due to the misreporting. For this study, we pool together the last two waves of AVQ administrated before COVID-19 outbreak (2018, 2019), and we restrict our sample to mothers and fathers who have the oldest child aged 17 or younger living in the same household. The total sample consists of ∼13 000 mothers and fathers (∼7000 families). In our sample, we reach a share of 6.17% of children whose daily activities are limited or severely limited, reasonably in line with the Eurostat estimated prevalence.^7^

## Measures

### Child disability

Measuring child disability is a big challenge. While measurements of disabilities among older persons are well-established and accepted, there is no such agreement for child disability. In line with the bio-social approach which insists that disability should be understood as the result of the interaction between medical impairments and the barriers faced within the social environment which in turn leads to limitations in activities,^34^^,^^35^ disability is measured using the Global Activity Limitation Indicator (GALI). The question reads as follows: ‘For at least the past six months, to what extent has your child been limited because of a health problem in activities that people usually do?’. We consider disabled if the activities of the child have been either severely limited or limited but not severely. We use this as a dummy indicator to identify the presence of a disabled child in the household.

### Health measurements

The health and well-being outcome measures considered in this study were: (i) general level of health, (ii) mental health and (iii) life satisfaction. The general level of health derived from the Self-Rated Health five-point Likert scale measure ranging from ‘Very good’ to ‘Very bad’ (the distribution of the responses is reported in table 1). For interpretability of the results, we inverted the scale, the higher is the level of health, the better the general level of health of the respondent.

**Table 1:** Sample characteristics

	Entire sample	Fathers	Mothers
No. of respondents	12 988	6129	6859
Outcome variables			
Self-rated health (SRH)			
Very bad	0.31	0.36	0.26
Bad	1.72	1.55	1.88
Moderate	18.83	18.49	19.14
Good	61.09	61.33	60.87
Very good	18.05	18.27	17.85
Mental health (SF-36 0–100 score mean and SD)	69.84 (16.90)	71.05 (16.57)	68.75 (17.13)
Well-being (life satisfaction 0–10 score, mean and SD)	7.40 (1.55)	7.39 (1.57)	7.41 (1.53)
Main independent variable			
Having a disabled child	6.17	6.07	6.25
Variables of interest			
Gender of the respondent			
Male	47.19	–	–
Female	52.81	–	–
Level of education			
Low level of education	29.90	34.48	25.82
High level of education	70.10	65.52	74.18
Control variables			
Age (years)			
Up to 34	16.52	11.42	21.07
35–44	48.41	45.06	51.41
45–54	31.31	37.20	26.07
Above 54	3.77	6.31	1.49
Year of interview			
2018	51.71	51.85	51.58
2019	48.29	48.15	48.42
Region of residence			
North-West	21.86	21.62	22.07
North-East	22.41	22.50	22.34
Centre	18.75	18.70	18.79
South	27.06	27.12	27.00
Islands	9.92	10.07	9.00

Notes: Values are given in percent. In the multivariate regression model, we used a dichotomized version of SRH: bad/very bad vs. very good/good/moderate.

**Table 2:** The link between having a disabled child and parental general level of health (0–4 self-rated health measure), mental health (standardized measure, beta coefficient) and well-being (standardized measure—life satisfaction score, beta coefficient reported)

	(1)	(2)	(3)
Variables	General level of health	Mental health (standardised)	Well-being (standardised)
Having a child with disability	−0.149***	−0.108**	−0.0739^+^
	[−0.202; −0.0949]	[−0.186; −0.0294]	[−0.161; 0.0131]
Controls	Yes	Yes	Yes
Observations	12 988	12 988	12 788

Notes: Multivariate linear regression models. Each column refers to a different linear regression. General level of health is 0–4 Likert scale based on the Self-Rated Health Measure. Higher level means better level of health. Mental health outcome is a 0–100 score based on the 5 items of SF-36: emotional wellbeing score. Higher is the score, better level of mental health is. The outcome has been standardized for interpretability. Well-being is measured via life satisfaction 0–10 Likert scale. Higher level means better life satisfaction. Outcome standardized for interpretability. All models control for gender of the respondent, age (up to 34, 35–44, 45–54, more than 55), level of education (low or high educational level), year of interview (2018, 2019), macro region of residence (North-West, North-East, Centre, South, Islands). Full regression table reported as Supplementary material; 95% confidence intervals are reported in brackets.

*** *P* < 0.001,

** *P* < 0.01,

* *P* < 0.05,

+ *P* < 0.10.

The mental health measure was derived from the Short Form (SF-36) Health Survey.^36^ SF-36 is a 36-item questionnaire on physical, mental and emotional health. In this study, we focused on the subscale (five items) that assesses mental health. The score is a continuous variable ranging from 0 to 100, with a higher score translating into better mental health. On average, the mean SF-36 was 68.84 with a SD of 16.90.

The well-being of the respondent is assessed using a single life satisfaction item: a scale from 0 to 10, with a higher score meaning a higher level of satisfaction. On average in our sample, the mean level of well-being was 7.40 with a SD of 1.55. Due to the skewness of the SF-36 mental health score and well-being and for interpretability purposes, we standardized the two scores.

### Socio-demographic characteristics and control variables

We aim to study to what extent the socio-economic status (measured via the level of parent’s education) is a protective factor on parental health even among parents of a disabled child. The original education variable is in four categories: university degree or above (ISCED 4+: 23%); high school diploma (ISCED 3: 47%); lower secondary education (ISCED 2: 26%); below lower secondary education (ISCED 0 and 1: 2%). Due to the distribution of the variable and considering the total number of cases, we decided to dichotomized into high level of education (ISCED 3+) vs. low level (ISCED 0–2).

We moreover control for a set of characteristics that might affect the health status of the respondent: gender and age of respondent (categorical: up to age 34 years, 35–44 years, 45–54 years, more than 55 years); area of residence (categorical variable: North West, North East, Centre, South, Islands) to account for the geographical differences; the year of the interview since we pooled two waves of data (categorical variable: being interviewed in 2018 or 2019).

## Methods

**Figure 1:**
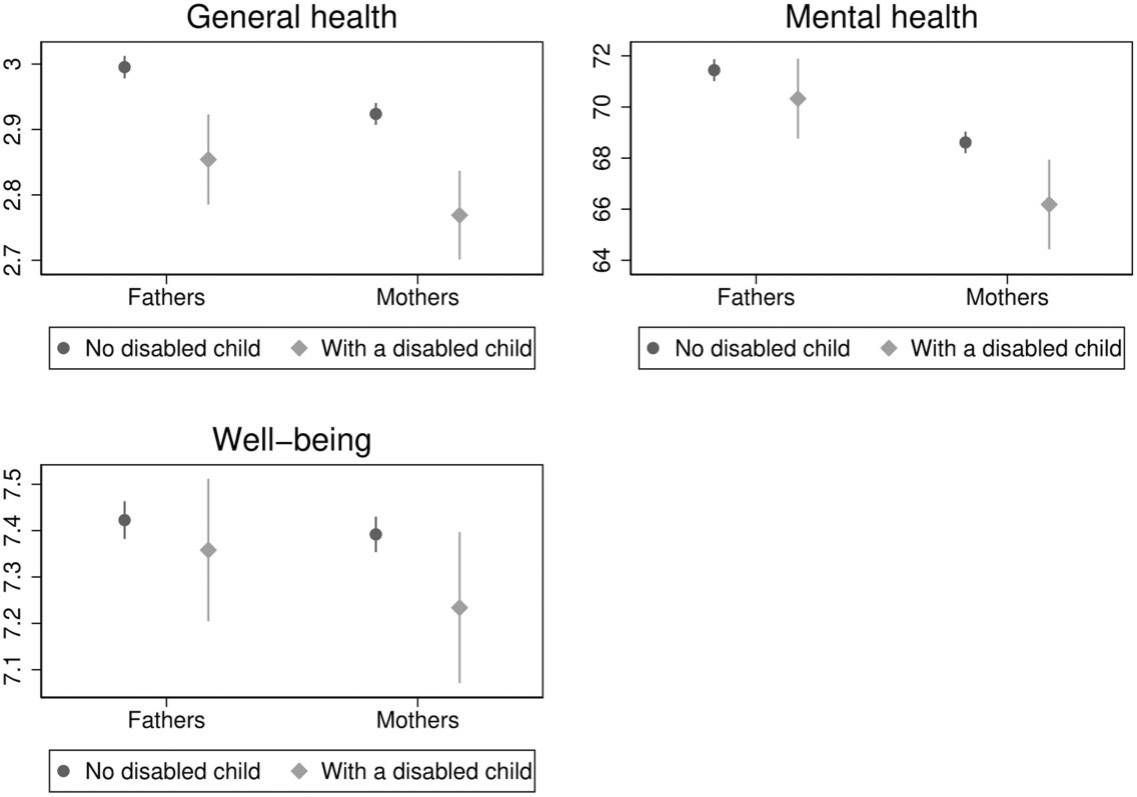
Predicted levels of general health (top left panel), (SF-36 score) mental health (top right panel) and well-being (life satisfaction score, bottom panel) for fathers and mothers having or not a child with disability. Notes: Predicted probabilities calculated on the basis of linear regression models with an interaction term between gender of respondent and having or not a disabled child. General level of health is 0–4 self-rated health measure, mental health derives from 0 to 100 SF-36 score and well-being from 0 to 10 life satisfaction score

**Figure 2:**
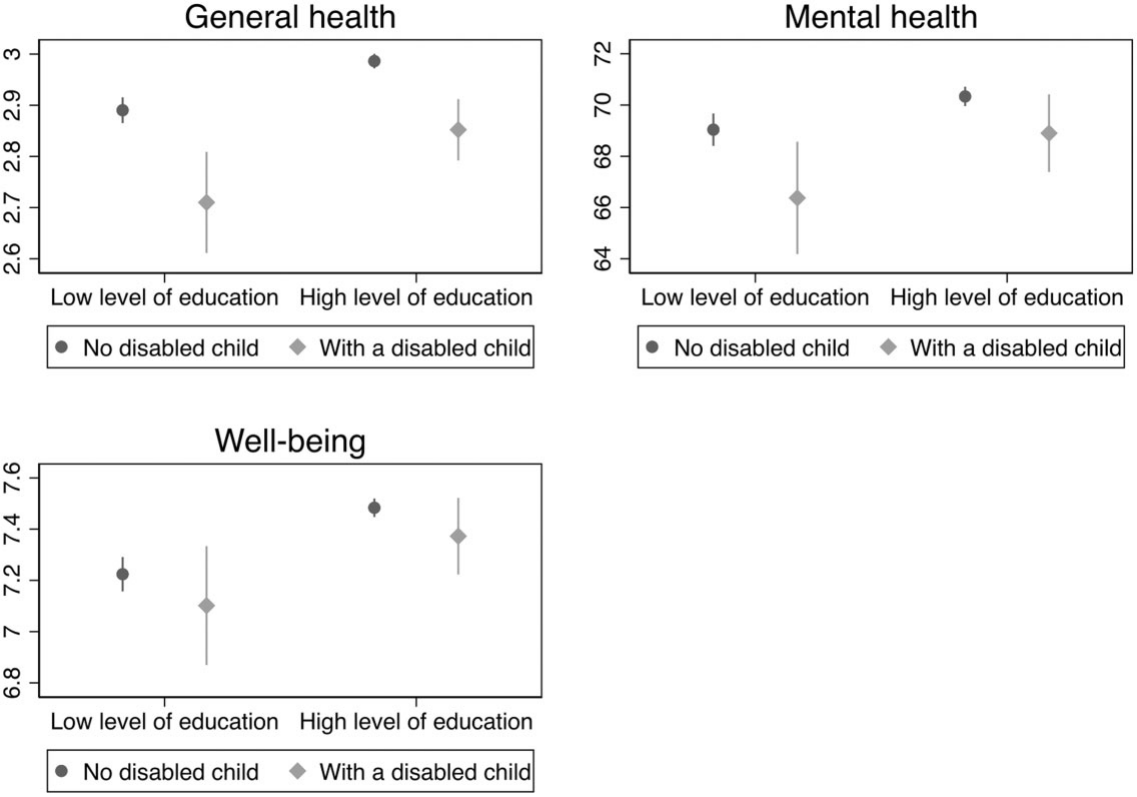
Predicted levels of general health (top left panel), (SF-36 score) mental health (top right panel) and well-being (life satisfaction score, bottom panel) by level of education for mothers and fathers having or not a child with disability. Notes: Predicted probabilities calculated on the basis of linear regression models with an interaction term between level of education and having or not a disabled child. General level of health is 0–4 self-rated health measure, mental health derives from 0 to 100 SF-36 score and well-being from 0 to 10 life satisfaction score

We employ a multivariate linear regression model approach to estimate the link between child disability and parental health. Separate linear regression models are run for each outcome: general level of health (0–4 Likert scale higher levels mean better health); SF-36 mental health score and well-being. For interpretability, we standardized the latter two outcomes. Since both parents might be interviewed and we might have a mutual influence across partners within a couple, the SEs were clustered at the household level. To estimate parental health and well-being disparities due to child disability, we report and comment on the estimated probabilities by gender of the parent (figure 1) and by his/her level of education (figure 2) after interacting the dummy variables of interests.

## Results

Results from bivariate associations (Supplementary table S1) show that having a disabled child living in the household is associated with lower level of general health (*P* values of ANOVA test <0.001). For instance, we observe that 3.25% of respondents with a disabled child report to be in poor SRH again against only 1.95% of their counterparts (*P* values of chi-squared test of 0.012). Having a disabled child is associated as well with lower levels of mental health (SF-36 mean score of 67.95 against 69.96; *P* values ANOVA test of differences across groups of 0.001) and lower well-being (7.29 vs. 7.41; *P* values of ANOVA test <0.05).

Estimates from the multivariate regression models (table 2) confirm the results of the bivariate associations. Controlling for gender, age, level of education and area of residence, we observe a negative relationship between child disability and all the health and well-being dimensions considered in the study, especially in terms of mental health. Having a disabled child is associated with a reduction: (i) in mental health equal to 11% of a SD (beta coefficient on standardized SF-36 score of −0.108, 95% CI [−0.186; −0.029]); (ii) in parental well-being equal to 8% of a SD (beta coefficient on standardized life satisfaction score of −0.074, 95% CI [−0.161; 0.013]) and (iii) and in general level of health (beta coefficient of −0.149, 95% CI [−0.202; −0.095] on 0–4 self-rated health scale). Such associations are clearly relevant, considering that with respect to general health the coefficient estimated for child disability is almost two times higher than the one calculated for the gender of the respondent, whereas in terms of mental health the reduction relative to have a disabled child is higher (in absolute terms) with respect to the protective factor of being highly educated. Similar results for general health are observed using a logit model with dichotomized version of general health (being in poor self-rated health or not, odds ratio of 1.525).

Turning to gender differences, we estimated for each outcome a regression model with an interaction term between having a disabled child and gender of the parent, and then report the predicted probabilities.

As shown in figure 1, child disability is negatively associated with the health and well-being of both parents, but the association is stronger for mothers, more likely the main caregivers, in particular in terms of mental health. Comparing those with and without a disabled child, the predicted SF-36 score (0–100 score) declined by −2.43 points among mothers—from 68.62 to 66.19—and only −1.12 points among fathers (from 71.44 to 70.32). Similarly, the estimated decline in levels of well-being (0–10 scale) was stronger among mothers (from 7.39 to 7.23, an estimated decline of −0.158 points) rather than fathers (decline of −0.064). We observe slightly less strong gender differences in the general level of health with a reduction observed on the 0–4 scale of SRH health for those with a disabled child with respect to a parent without a disabled child of −0.16 points among mothers and of −0.14 among fathers.

Relevant socio-economic differences in the association between child disability and parental health and well-being are observed (figure 2). Low-educated parents report a stronger, negative, influence of having a disabled child. Among those with a disabled child, an estimated reduction of −0.18 points in the general level of health (over 0–4 scale), of −2.66 in SF-36 mental health score and of −0.122 in 0–10 life satisfaction scale is observed among parents with a low level of education vs. respectively a reduction of −0.134, −1.434 and −0.111 points for high SES parents.

## Discussion

This study aimed at documenting the importance of child disability as a neglected source of health disadvantage for parents and a shaping factor in their lives. We showed that parents of disabled children have worse self-rated and mental health, and lower life satisfaction. We moreover found that health disparities due to child disability are particularly relevant for mothers and low SES families. These are the social groups that are more vulnerable to this condition, either because they have to bear the higher practical and emotional costs, as main caregivers (mothers), or because they lack the necessary resources to cope with the relative economic, social and bureaucratic challenges (low SES parents). In both cases, as main caregiver, or in absence of enough material and cultural resources to (even partially) outsource the care of the disabled child, an individual’s life is fully shaped by the child disability, which implies higher stress, stigma, lack of personal and leisure time, worse work-family balance. While bringing solid and novel evidence of child disability as a potential source of disadvantage for parental health, this work is not without limitations: having access only to cross-sectional data, the results should be interpreted in associational terms. We cannot rule out, for instance, selection issues in having a disabled child, that may lead to reverse causality. Moreover, we have no information on whether the disability is permanent or temporary, and about its onset.

Our findings provide evidence that children’s characteristics are non-negligible social stratification factors for parents’ health, likely shaping their life opportunities, conditions and behaviours. Further research should more systematically adopt this perspective (i.e. from children to parents) to study the impact of child conditions (beyond disability) on other parental health outcomes. The paradigm that considers child disability as a family issue should also be applied to investigate spillover effects on other family members’ health, such as siblings and grandparents. What constrained us from doing that is the lack of suitable data. Therefore, we claim the importance of including measures of child disability in panel data with a multi-actor design. This would be the first essential step to provide systematic evidence of the branched-out effects of a child’s disability on the other family members, in several different life dimensions. Ideally, we should be able to also measure type and severity of a child disability to ultimately distinguish disability-specific dynamics from universal family needs, outcomes and challenges in the presence of child’s disability. That should support the development of comprehensive policy interventions for families, which are, of course, more cost-effective, and ideally flexible enough to have some disease-specific implementations. The design and implementation of suitable health interventions should be done taking into account the crucial role of health professionals in assessing the effect of child disability on family members’ health. More in general, policy interventions should be devoted to invest in family-centred care system able to support parents as part of a larger care-strategy for the disabled child, especially targeting mothers and low SES families.

## Supplementary Material

ckad168_Supplementary_Data

## Data Availability

Data used in this study are available upon request from Italian National Statistics Office ISTAT. To access the data, researchers need to register on ISTAT website and sign a confidentiality deed poll. Key points Child disability is an important source of health disadvantage for parents, who show worse mental health and lower general well-being. The health disadvantage associated to child disability is higher for mothers than for fathers, Parental education is a relevant protective factor against the negative consequences of child disability on parents’ health. Health disparities are multi-dimensional, stemming from the intersection of several social inequalities: disability, gender and socio-economic status. Family-centred care models should invest on supporting parents’ health as part of a larger care-strategy for the child with any form of disability.

## References

[1] Ferguson PM. Mapping the family: disability studies and the exploration of parental response to disability. In: Albrecht G, Seelman KD, Bury M, editors. Handbook of Disability Studies. Thousand Oaks: Sage, 2001: 373–95.

[2] Arora K , Wolf DA. Is there a trade-off between parent care and self-care? Demography 2014;51:1251–70. 24903840 10.1007/s13524-014-0309-6

[3] Pinquart M , Sörensen S. Differences between caregivers and noncaregivers in psychological health and physical health: a meta-analysis. Psychol Aging 2003;18:250–67. 12825775 10.1037/0882-7974.18.2.250

[4] Schulz R , Sherwood PR. Physical and mental health effects of family caregiving. J Soc Work Educ 2008;44:105–13. 10.1097/01.NAJ.0000336406.45248.4c PMC2791523 18797217

[5] Vitaliano PP , Zhang J, Scanlan JM. Is caregiving hazardous to one’s physical health? A meta-analysis. Psychol Bull 2003;129:946–72. 14599289 10.1037/0033-2909.129.6.946

[6] United Nations Children’s Fund. Seen, Counted, Included: Using Data to Shed Light on the Well-Being of Children with Disabilities. New York: UNICEF, 2021.

[7] Eurostat. Children with Activity Limitation due to Health Problems. Bruxelles: European Commission, Eurostat, 2017.

[8] European Commission. Access to Quality Education for Children with Special Educational Needs. Bruxelles: European Commission, 2018.

[9] Capitani E , Lorenzini C, Biuzzi A, et al Factors influencing the first thousand days of life. Eur J Public Health 2022;32:ckac129.671.

[10] Lee DS , Cederbaum JA, Davis JP, et al Maternal and adolescent depressive symptoms and family conflict: an autoregressive cross-lagged examination of competing models in multi-stressed mothers and adolescents. Fam Process 2023;62:254–71. 35545438 10.1111/famp.12779

[11] Zhou X , Sun X. Family depression profiles among adolescents and their parents: a group-based multitrajectory modeling. J Fam Psychol 2021;35:886–96. 33871268 10.1037/fam0000849

[12] Kalmijn M , De Graaf PM. Life course changes of children and well-being of parents. J Marriage Fam 2012;74:269–80.

[13] Hogan D , Msall ME, Goldscheider FK, et al Family Consequences of Children’s Disabilities. New York: Russell Sage Foundation, 2012.

[14] Stabile M , Allin S. The economic costs of childhood disability. Future Child 2012;22:65–96. 22550686 10.1353/foc.2012.0008

[15] Burton P , Lethbridge LN, Phipps S. Mothering children with disabilities and chronic conditions: long-term implications for self-reported health. Can Public Policy 2008;34:359–78.

[16] Marquis SM , McGrail K, Hayes MV. A population-level study of the mental health of siblings of children who have a developmental disability. SSM Popul Health 2019;8:100441. 31334325 10.1016/j.ssmph.2019.100441 PMC6617296

[17] Brekke I , Nadim M. Gendered effects of intensified care burdens: employment and sickness absence in families with chronically sick or disabled children in Norway. Work Employ Soc 2017;31:391–408.

[18] Kvist AP , Nielsen HS, Simonsen M. The importance of children’s ADHD for parents’ relationship stability and labor supply. Soc Sci Med 2013;88:30–8. 23702207 10.1016/j.socscimed.2013.04.001

[19] Vinck J , Van Lancker W. An intersectional approach towards parental employment in families with a child with a disability: the case of Belgium. Work Employ Soc 2020;34:228–61.

[20] Craig L , Mullan K. Parenthood, gender and work-family time in USA, Australia, Italy, France and Denmark. J Marriage Fam 2010;72:1344–61.

[21] Damaske S. Gender, family, and healthcare during unemployment: healthcare seeking, healthcare work, and self-sacrifice. J Marriage Fam 2022;84:291–309. 35450385 10.1111/jomf.12801 PMC9017794

[22] Bixby LE. Disability is not a burden: the relationship between early childhood disability and maternal health depends on family socioeconomic status. J Health Soc Behav 2023;64:354–69. 37097010 10.1177/00221465231167560 PMC10486143

[23] Olsson MB , Hwang CP. Well-being, involvement in paid work and division of child-care in parents of children with intellectual disabilities in Sweden. J Intellect Disabil Res 2006;50:963–9. 17100956 10.1111/j.1365-2788.2006.00930.x

[24] Horsley S , Oliver C. Positive impact and its relationship to well-being in parents of children with intellectual disability: a literature review. Int J Dev Disabil 2015;61:1–19.

[25] Boyd BA. Examining the relationship between stress and lack of social support in mothers of children with autism. Focus Autism Other Dev Disabil 2002;17:208–15.

[26] Hastings RP. Parental stress and behaviour problems of children with developmental disability. J Intell Dev Disabil 2002;27:149–60.

[27] Findler L. The experience of stress and personal growth among grandparents of children with and without intellectual disability. Ment Retard 2014;52:32–48. 10.1352/1934-9556-52.1.32 24635690

[28] Levine KA. Against all odds: resilience in single mothers of children with disabilities. Soc Work Health Care 2009;48:402–19. 19396709 10.1080/00981380802605781

[29] Chetty S , Friedman AR, Taravosh-Lahn K, et al Stress and glucocorticoids promote oligodendrogenesis in the adult hippocampus. Mol Psychiatry 2014;19:1275–83. 24514565 10.1038/mp.2013.190 PMC4128957

[30] Albrecht GL , Devlieger PJ. The disability paradox: high quality of life against all odds. Soc Sci Med 1999;48:977–88. 10390038 10.1016/s0277-9536(98)00411-0

[31] Olsson MB , Hwang CP. Depression in mothers and fathers of children with intellectual disability. J Intellect Disabil Res 2001;45:535–43. 11737541 10.1046/j.1365-2788.2001.00372.x

[32] Drew JAR. Disability, poverty, and material hardship since the passage of the ADA. Disabil Stud Q 2015;35:4947. 27042381 10.18061/dsq.v35i3.4947 PMC4812446

[33] Cohen PN , Petrescu-Prahova M. Gendered living arrangements among children with disabilities. J Marriage Fam 2006;68:630–8.

[34] Oliver M. Social Work with Disabled People. Basingstoke: Macmillan, 1983.

[35] World Health Organization. Classification of Functioning, Disability and Health: Children and Youth Version. Geneva: WHO, 2007.

[36] Ware JE Jr , Sherbourne CD. The MOS 36-item short form health survey (SF-36). I. Conceptual framework and item selection. Med Care 1992;30:473–83. 1593914

