# Supplementary material for: Child disability as a family issue: a study on mothers’ and fathers’ health in Italy
Source: Eur J Public Health. 2023 Oct 5;34(1):79–84. doi: 10.1093/eurpub/ckad168 (PMC10843958; doi:10.1093/eurpub/ckad168)
Supplement: ckad168_Supplementary_Data [file ckad168_supplementary_data.docx]

**SUPPLEMENTARY MATERIALS**

**Table S1** Bivariate Associations – p-value of ANOVA test

|  | Family with a disabled child | Family with no disabled child | P-value |
| --- | --- | --- | --- |
| General level health | 2.78 | 2.96 | <.001 |
| Mental health (0-100) | 67.95 | 69.96 | .001 |
| Well-being (0-10) | 7.29 | 7.41 | .035 |

Note: General level of health is derived from 0-4 Likert Self-Rated Health measure (higher levels mean better level of health). Mental health is derived from SF-36 score and well-being measure is derived from life satisfaction (0-10 score).

**Table S2.** The association between having a disabled child and parental health outcomes: general level of health, mental health (SF36 score – standardized), and well-being (standardized). Multivariate linear regression model

|  | General health | Mental health | Well-being |
| --- | --- | --- | --- |
| VARIABLES | 0-4 self-rated health score | SF-36 score standardised | Life Satisfaction score standardised |
|  |  |  |  |
| Having a disabled child | -0.149*** | -0.108** | -0.074+ |
|  | (0.817 - 0.909) | (-0.186 - -0.029) | (-0.161 - 0.013) |
| Gender of the respondent (Woman) | -0.072*** | -0.172*** | -0.024+ |
|  | (-0.094 - -0.050) | (-0.203 - -0.141) | (-0.050 - 0.003) |
| Having a high level of education | 0.099*** | 0.081*** | 0.168*** |
|  | (0.071 - 0.127) | (0.040 - 0.123) | (0.122 - 0.214) |
| Age of the respondent (Ref below 35) |  |  |  |
| 35-44 yo | -0.180*** | -0.106*** | -0.0623* |
|  | (-0.212 - -0.148) | (-0.155 - -0.056) | (-0.116 - -0.009) |
| 45-54 yo | -0.322*** | -0.181*** | -0.116*** |
|  | (-0.358 - -0.286) | (-0.236 - -0.126) | (-0.175 - -0.056) |
| More than 55 | -0.523*** | -0.313*** | -0.223*** |
|  | (-0.596 - -0.451) | (-0.418 - -0.208) | (-0.338 - -0.109) |
| Year of interview 2019 (Ref 2018) | -0.008 | 0.0713*** | 0.052* |
|  | (-0.033 - 0.016) | (0.033 - 0.109) | (0.010 - 0.094) |
| Region of residence (Ref North-West) |  |  |  |
| North-East | 0.071*** | 0.0393 | 0.048 |
|  | (0.034 - 0.107) | (-0.017 - 0.095) | (-0.013 - 0.108) |
| Center | 0.034+ | -0.00917 | -0.053 |
|  | (-0.0033 - 0.072) | (-0.068 - 0.050) | (-0.117 - 0.011) |
| South | 0.0133 | -0.010 | -0.125*** |
|  | (-0.022 - 0.049) | (-0.066 - 0.045) | (-0.186 - -0.064) |
| Islands | 0.051* | 0.048 | -0.070+ |
|  | (0.0017 - 0.099) | (-0.025 - 0.121) | (-0.152 - 0.0125) |
| Constant | 3.107*** | 0.116*** | -0.011 |
|  | (3.063 - 3.151) | (0.048 - 0.185) | (-0.086 - 0.063) |
|  |  |  |  |
| Observations | 12,988 | 12,988 | 12,788 |
| R-squared | 0.041 | 0.013 | 0.014 |
| Confidence intervals reported in parentheses |  |  |  |
| *** p<0.001, ** p<0.01, * p<0.05, + p<0.10 |  |  |  |
